# Differential effects of peritoneal and hemodialysis on circulating regulatory T cells one month post initiation of renal replacement therapy 

**DOI:** 10.5414/CN110158

**Published:** 2020-10-19

**Authors:** Carlotta Caprara, Valentina Corradi, Elisa Scalzotto, Anna C. Frigo, Marta Proglio, Rahul Sharma, Claudio Ronco

**Affiliations:** 1 International Renal Research Institute of Vicenza,; 2 Department of Nephrology, Dialysis and Transplantation, St. Bortolo Hospital, Vicenza,; 3 Biostatistics, Epidemiology and Public Health Unit, Department of Cardiac, Thoracic and Vascular Sciences, University of Padova,; 4 Department of Medicine, University of Padova, Padova, Italy, and; 5 Center for Immunity, Inflammation and Regenerative Medicine, Division of Nephrology Department of Medicine, University of Virginia, Charlottesville, VA, USA; * Dr. Rahul Sharma co-senior author on the manuscript

**Keywords:** Treg, FOXP3, CD25, dialysis, RRT

## Abstract

Backgroundː Chronic kidney disease stage G5 (CKD G5) patients show an activated but impaired immune system. One function of the FOXP3^+^ regulatory T (Treg) cells is to preserve tolerance to self while maintaining the ability to fight infectious agents. The aim of this pilot study is to evaluate longitudinal changes in Treg cells before and 1 month after the first dialysis treatment. Materials and methodsː CKD G5 patients not yet on dialysis were enrolled and started on hemodialysis (HD) or peritoneal dialysis (PD). Tregs were analyzed by flow cytometry at two time points: T0 (before the first dialysis treatment) and T1 (1 month after the first dialysis session). Wilcoxon test for dependent samples was used to compare the mean percentage difference between T0 and T1: Δ% = 100 × [(T1 – T0) / T0]. Resultsː 21 patients were enrolled: 8 on HD and 13 on PD. The proportion of total lymphocytes (low side scatter lymphocyte gate) and T lymphocytes (in the CD3^+^CD4^+^ gate) did not change significantly 1 month after the start of dialysis in both groups. Treg cells (as CD25^+^FOXP3^+^, FOXP3^+^, or CD25^+^CD127^–^), analyzed as percentage of the lymphocyte gate, showed a significant increase post PD (CD25^+^FOXP3^+^: median = 35.92; p = 0.0425; FOXP3^+^: median = 30.85; p = 0.0479 and CD25^+^CD127^–^: median = 23.71; p = 0.0215). The same populations, did not change 1 month after the first dialysis session. Conclusionː Our study is the first to evaluate longitudinal effects of dialysis on Treg cells in uremia and suggests that PD was more effective in increasing Treg levels 1 month post initiation of dialysis and may contribute to improvement of inflammatory status. Thus, PD may contribute to better outcomes for patients with renal dysfunction, also maintaining homeostasis of peritoneal and renal tissues.

## Introduction 

One of the major challenges of the immune system is to preserve immune tolerance to self while maintaining the ability to fight foreign pathogens and infectious agents [1]. Several studies have demonstrated that patients with chronic kidney disease (CKD) stage G5 who are not on dialysis (CKD G5), CKD patients stage G5 on dialysis (CKD G5D), or patients with acute kidney injury [2] suffer from immune system dysregulation as characterized by immune incompetence and signs of chronic inflammation [3]. 

Inadequate responsiveness to vaccination, e.g., hepatitis B, was described in patients with end-stage renal disease (ESRD) [4]. These patients are also more susceptible to viral as well as bacterial infections, which are the second leading cause of mortality (after cardiovascular) during renal replacement therapy [5]. Uremia has also been reported to influence proinflammatory cytokine levels [6]. An additional explanation for this state of immune dysregulation may be the altered number or impaired function of the regulatory T cells (Treg) [7]. 

Treg cells are a subset of CD4 T cells that highly express the high-affinity IL-2 receptor α (CD25) and the lineage-defining transcription factor forkhead box P3 (FOXP3) with low or absent expression of CD127 [8]. In addition to their widely known function to inhibit the activation of CD4 and CD8 T cells and antigen presenting cells, Treg cells also inhibit innate immune cells such as monocytes/macrophages and neutrophils [9, 10]. Treg cells constitute a natural anti-inflammatory mechanism, and thus their modulation may be a key determinant of the immune status in acute and chronic kidney diseases [11]. Further, modulation of Treg numbers up or down leads to resistance or increased susceptibility to kidney injury, respectively [12]. 

T helper (Th) dysregulation is strongly associated with long term uremia especially in patients displaying cardiovascular complications [13]. Renal replacement therapy has been shown to influence this dysregulation as compared to healthy controls [14]. 

While immune defects are well described, it is still not clear whether the type of dialysis modality (hemodialysis (HD) versus peritoneal dialysis (PD)) differentially affects immunocompetence [15]. The majority of published studies have compared patients with healthy controls. The aim of this pilot study is to evaluate the longitudinal changes in the number of Treg cells before and 1 month after the first dialysis session. We focused on the impact of HD and PD on the circulating levels of Tregs in patients. 

## Materials and methods 

### Ethics committee approval 

The study was conducted in compliance with the Declaration of Helsinki. All participants gave informed written consent, and the study protocol was approved by of San Bortolo Hospital of Vicenza Ethics Committee, chairperson Francesco Salsa, Vicenza, Italy (Experimentation N.58/14) on December 29, 2014. 

### Study population 

From February 2015 to February 2016, a cross-sectional pilot study was conducted in International Renal Research Institute of Vicenza (IRRIV) with the aim to evaluate the influence of dialysis treatment (HD or PD) on Treg cells. Peripheral blood samples were obtained from CKD G5 patients (Table 1) who were not yet on dialysis (eGFR < 15 mL/min/1.73m^2^). Patients who needed continuous renal replacement therapy (CRRT) in the previous 3 months, had received a transplant, had an autoimmune diseases, lymphoma, or leukemia, were on immunosuppressive drugs, had undergone thymectomy, had immune deficiency or myasthenic diseases were not included in the study. Personal and treatment data were also recorded. The blood samples were drawn at the time of admission, before starting dialysis; this time point was called T0. The patients were on regular PD or HD for 1 month and the blood samples were drawn again 1 month after the start of dialysis, just before dialysis was started on that day; this time point was termed T1. 

### Isolation of peripheral blood lymphocytes 

Peripheral blood mononuclear cells (PBMC) were freshly isolated from 49 mL of heparinized peripheral blood by density gradient centrifugation using Ficoll-Paque PLUS (GE Healthcare, Chicago, IL, USA). PBMC were collected from the interphase, washed twice in PBS (1×) and resuspended in complete medium (RPMI 1640 Medium, GlutaMAX Supplement (supplemented with L-alanyl-L-glutamine dipeptide (Gibco, ThermoFisher Scientific, Waltham, MA, USA), 100 IU/mL penicillin, 100 µg/mL streptomycin (Penicillin-Streptomycin, Sigma Aldrich, St. Louis, MO, USA), and 10% pooled human serum (Human Serum Type AB male, EuroClone, Milan, Italy). An aliquot containing 0.5 to 1 million PBMC was frozen in RPMI 20% human serum, 10% DMSO (Sigma Aldrich) at –80 °C. 

### Flow cytometry analysis 

**Figure 1.**
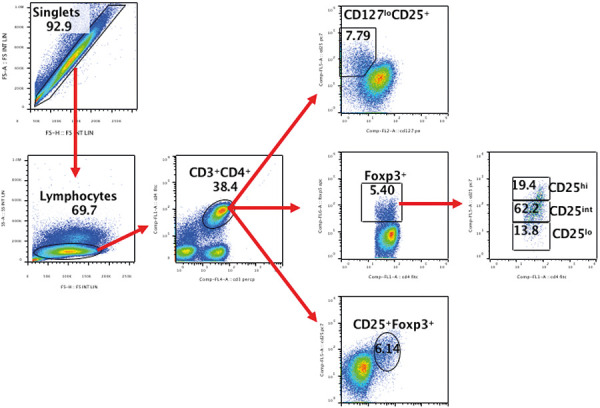
Gating strategy for analysis of peripheral blood Treg cells.

Cytometric analysis was performed according to standard procedures, and samples were acquired on a Navios Flow Cytometer (Beckman Coulter, Brea, CA, USA) followed by data analysis using Kaluza Flow Cytometry Analysis Software (Beckman Coulter) and FlowJo^TM^ (FlowJo Inc., BD, Franklin, NJ, USA). Cryopreserved PBMCs were thawed and analyzed as follows: A total of 0.5 – 1 million PBMC in 100 µL of complete medium were incubated for 30 minutes at room temperature with 5 µL anti-human CD3-PerCP (BioLegend, San Jose, CA, USA), 5 µL anti-human CD4-FITC (BioLegend), 5 µL anti-human CD25-PECy7 (BioLegend), and 20 µL anti-human CD127-PE (Beckman Coulter) antibodies. After washing with 1X PBS, cells were incubated over night at 4 °C with the 1× FIX/PERM buffer of the internalization kit (eBioscience, Thermofisher) following manufacturer’s instructions. The next day, cells were washed and then incubated for 30 minutes at 4 °C with 5 µL of anti-human FOXP3-APC antibody (eBioscience). After washing with the washing buffer, the PBMCs were analyzed with FACS, with at least 20,000 lymphocyte-gated events acquired for each sample. Mean fluorescence intensity (MFI) was determined using the geometric mean value of the respective marker antibody. The cell populations were analyzed either as % of CD3^+^CD4^+^ (T lymphocytes) or as % of lymphocytes in the live side scatter low gate (Figure 1). 

### Statistics 

Continuous variables were expressed as median and interquartile range (IQR) or mean ± standard deviation (SD) according to their distribution. The categorical variables were described as proportions. To compare the different populations, we considered the “delta” of value between the final value (1 month after the initiation of first dialysis) – T1 – and the “predialysis” value – T0. The percentage was calculated as Δ% = 100 × [(T1 – T0) / T0]. Wilcoxon test was applied to compare the mean Δ% difference between T0 and T1. A p-value of p < 0.05 was considered significant. 

## Results 

### Cohort description 

Peripheral blood samples were obtained from 21 CKD G5 patients (Table 1) not yet on dialysis. Of them, 8 were started on HD (N = 8), and 13 were started on PD (N = 13). The total cohort (8 HD + 13 PD, n = 21) included 87.5% males in the HD group and 84.6% males in the PD group. The mean age was 68 years in the HD group and 67 years in the PD group. The characteristics of the participants in the two groups are shown in Table 1. All patients had hypertension as the primary or secondary cause. Approximately 75% of HD patients and 46% of PD patients had diabetes. The cause of CKD was divided into 3 groups as shown in Table 1. The comorbidities were categorized on the basis of vasculopathy of different origin and described as cerebral, cardiac, and peripheral vasculopathies. None of the patients was diagnosed with any infections, nor was any incidence of peritonitis found in the PD patients. All PD patients were on continuous ambulatory peritoneal dialysis (CAPD) treatment; 46.2% used twin-bag Physioneal 40 LPB 5262G (Baxter, Castlebar, Ireland); 53.8% used BicaVera 2536701 (Fresenius Medical Care, Deutschland GmbH, Bad Hamburg, Germany). Among the HD patients, 37.5% had AV fistula; 50% had jugular catheter, and 12.5% had graft. As for the HD modality, 62.5% were on bicarbonate dialysis and 37.5% were on high-flux dialysis (HFD). Although 2 of the 13 PD and 1 of the 8 HD patients were on antibiotics during enrolment, the drug course ended before the start of the study and none of the patients had an active infection, peritonitis, or received antibiotic treatment during the course of the study. 

### Lymphocyte proportions in HD and PD patients 

The proportion of lymphocytes (gated on forward scatter (FSC) vs. side scatter (SSC) (Figure 1) and T lymphocytes (analyzed as CD4^+^CD3^+^ in the lymphocytes gate; data not shown) did not change before and after the dialysis treatment (as evaluated 1 month after the start of dialysis) in PD and HD patients. Similar to the total lymphocytes, we did not observe any change in the proportion of CD4^+^ T cells in the peripheral blood of HD and PD patients during this time period. 

### PD correlates with an increase in the proportion of Tregs in gated lymphocytes 

**Figure 2.**
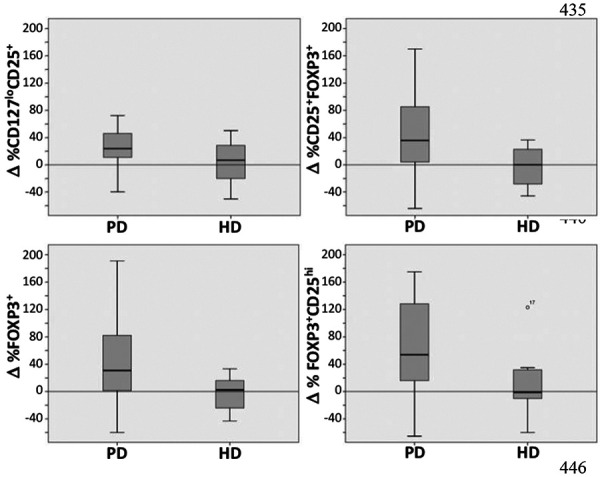
Changes in the proportions of Treg cells (Δ%) before dialysis and 1 month after first dialysis in (a) CD25^+^CD127^–^, (b) CD25^+^FOXP3^+^, (c) FOXP3^+^, and (d) CD25^hi^ gated on FOXP3^+^ cells populations.

Since literature survey indicates diverse approaches of evaluating Tregs in the peripheral blood, we analyzed the proportion of Tregs in three different ways by analyzing the difference in Treg percentages (Δ%) measured before and 1 month after the initiation of dialysis as (a) CD25^+^FOXP3^+^ (b) CD25^+^CD127^–^, and (c) FOXP3^+^ populations [16] and calculated as Δ (see Materials and Methods for details). As shown in Table 2 and Figure 2, we observed a significant increase in the proportion of Tregs in the CD3^+^CD4^+^ gated peripheral blood lymphocytes in patients undergoing PD as indicated by statistically significant changes in the Δ% in the Tregs in all three populations. For further analysis, we divided the CD4^+^FOXP3^+^ cells into CD25^lo^, CD25^int^, and CD25^hi^ subpopulations. Interestingly, the change was only observed in the CD25^hi^ subpopulation and not the CD25^lo^ and CD25^int^ subsets. The same cellular populations did not change when measured 1 month after the first dialysis session in HD patients (Table 2). 

### Changes in CD25 expression in HD vs. PD patients 

CD25 also serves as an indicator of cellular activation, therefore, we characterized CD25 expression on a cell-intrinsic basis by analyzing the MFI of CD25. The Δ% in CD25 MFI after 1 month of HD dialysis showed a statistically significant increase in CD25^+^FOXP3^+^ (median 4.10; 95% CI 3.26 – 25.80; p = 0.0078) population as well as in the subpopulations of FOXP3^+^ divided for CD25, CD25^int^ (median 3.59; 95% CI 0.95 – 19.42; p = 0.0156), a decrease that did not reach significance in CD25^lo^ (median –4.32; 95% CI –9.09 – 4.69; p = 0.0547) populations, and no changes in CD25^hi^ population. We found similar results for PD patients where we observed a statistically significant increase in CD25 expression in the subpopulations of CD25^+^FOXP3^+^ divided into CD25^int^ (median 4.69; 95% CI 2.58 – 14.52; p = 0.0398) but not in CD25^lo^ (median –8.51; 95% CI –12.95 – 0.78; p = 0.0046) that instead showed a significant decrease. We did not find significant changes in CD25 expression in other cell populations (data not shown). 

### Changes in FOXP3 expression between HD vs. PD patients 

We also analyzed the MFI of FOXP3 as a change (Δ%) before and 1 month after initiation of dialysis. The data shows that in patients with HD, a statistically significant decrease in FOXP3 MFI was observed in the lymphocyte-gated populations (median –14.14; 95% CI –31.46 – 4.05; p = 0.0234) and the T lymphocyte-gated populations (median –16.41; 95% CI –27.45 – 6.47; p = 0.0391). We did not observe any changes in FOXP3 MFI in other cell populations. In PD patients, we observed a statistically significant decrease in FOXP3 expression in CD25^+^FOXP3^+^ (median –2.14; 95% CI –8.58 – 0; p = 0.0161) and CD25^int^-gated subpopulation of FOXP3^+^ cells (median –3.59; 95% CI –5.07–2.48; p = 0.0327). No other significant changes in FOXP3 MFI were observed in any other cell populations (data not shown). 

## Discussion 

In this study we analyzed the effect of the initiation of dialysis treatment on the same patient longitudinally and compared the percentage of Treg in the same individual before and 1 month after the initiation of dialysis. Interestingly, the outcomes of PD or HD patients were quite different in that we observed a positive Δ% of Treg over 1 month in PD patients whereas no statistically significant changes were observed in HD patients. A possible reason for differences in the % change in PD and not in the HD group could be mobilization of cells from lymphoid compartments to circulation for eventual trafficking to the peritoneum to suppress potential inflammation in the peritoneum and elsewhere [17]. Recent experimental studies highlight that mice undergoing ischemia reperfusion surgery had fewer Tregs in the spleen, and the Treg levels were increased in the blood and injured kidneys, indicating Treg mobilization [18]. Although a greater number of HD patients had diabetes and there could be multiple other confounding factors, we did not observe a statistically significant effect and studied the patients longitudinally to hopefully minimize the effect of comorbidities on Treg levels. 

The higher Treg percentages may contribute to a lower systemic inflammatory status in these patients. Indeed, it was shown that an increase in the CD4^+^ cells in the peritoneal exudate of PD patients correlated with improved peritonitis outcomes and may be linked with the mobilization of lymphocytes [19]. Another possible explanation for this difference between PD and HD can be the higher biocompatibility of the peritoneum as compared to the HD lines and filter. Studies have shown that HD leads to an increase of the proinflammatory monocytes in the circulation [20, 21] as well as an increase in the granularity of the granulocytes [22]. The expression of activation markers CD11b and CD18 was also increased among the leukocytes in patients on HD [21, 22, 23], and it was suggested that chemical incompatibility of membranes may be involved in this immune activation [23]. The alteration in the Treg proportions may be a downstream effect of these changes in the innate immune cells. 

The increase primarily in the CD25^hi^ population and not the CD25^lo^ population of Tregs is also indicative of actively proliferating CD25^+^ Tregs [24], which may be beneficial, rather than the CD25^lo^ cells, which have recently been implicated in autoimmunity and inflammatory diseases [25]. We also considered the level of FOXP3 and CD25 expression on a per-cell basis by measuring the MFI of FOXP3 and CD25 in Treg cells. The MFI of CD25 was also significantly increased post dialysis in the CD25^int^ but not in the CD25^lo^ populations in the PD patients or the HD patients, indicating activation of Tregs. Interestingly, there was a decrease in the CD25 MFI in both PD and HD patients in CD25^lo^ populations (significant only in PD patients). We also observed a decrease in the FOXP3 MFI in the lymphocyte- and T lymphocyte-gated populations in HD patients. Kaul et al. [26] demonstrated that 6 weeks after initiation of intermittent HD, in vitro proliferation indices of T cells were increased significantly; however, no studies on Tregs were presented. Other studies have indicated alterations in Treg levels in HD [27], showing both increases [14] as well as decreases [28]. The majority of these studies compare patients with healthy controls. Comparing different groups of patients may lead to misinterpretation of results due to the inherent variability in the immune status in a diverse set of individuals. In our study, we compared the same patients longitudinally before and after their initiation of dialysis and the outcomes are internally controlled with dialysis being the only variable and a more defined effect of dialysis on Treg cells. Alternately, in measuring Tregs 1 month after dialysis, we may have missed the timeline for the Treg responses in HD. 

Tregs are involved, and are extremely important, in the maintenance of immune homeostasis [11] and a correct balance of Tregs and effector T cells being critical with Treg deficiency contributing to proinflammatory conditions and too high Tregs reflecting anergy and immune paralysis as observed in solid tumors [29]. Our data indicates that the elevated numbers of circulating Tregs after PD session may be beneficial by contributing to restore a T helper cell balance, which has been shown to be disrupted in peritoneal fluids leading to inflammation, fibrosis, and peritonitis [30]. 

## Conclusion 

In conclusion, our study is the first to evaluate the effect of PD and HD on the status of Treg cells to understand their role in immune homeostasis. PD was more effective in increasing Treg levels when analyzed 1 month post initiation of dialysis, which may contribute to improvement of inflammatory status. The results strongly imply that dialysis may be beneficial in restoring the Treg homeostasis as observed with increase in the activation status indicated by the higher MFI of CD25 in both PD and HD and an increase in proportion of Tregs in PD. 

## Funding 

We acknowledge the support of the non-profit organization FIDAS for the present publication. 

## Conflict of interest 

The authors declare that they have no conflict of interest. 

**Table 1.** Patient characteristics.

	PD	HD
Patients (F : M)	13 (2 : 11)	8 (1 : 7)
Mean age	67	68
Hypertension	13 (100%)	8 (100%)
Diabetes	6 (46%)	6 (75%)
Cause of CKD		
Nephropathy	4 (31%)	1 (12%)
Genetic	2 (15%)	1 (12%)
Secondary	7 (54%)	6 (75%)
Comorbidities (vasculopathy)		
Cerebral	0	4 (50%)
Cardiac	4 (31%)	5 (62%)
Peripheral	3 (23%)	3 (37%)

**Table 2.** Tabulated results of flow cytometry analysis in the peripheral blood of peritoneal dialysis and hemodialysis patients. The proportions of various Treg subsets were analyzed in three different ways as CD25+FOXP3+, CD127loCD25+, and FOXP3+ (further divided into CD25hi, CD25int, and CD25lo subpopulations) in the CD3+CD4+ population as % of lymphocyte gate as shown in Figure 1. The change in the Treg proportion (Δ%), calculated as described in the Materials and Methods section is displayed.

		Variable	N	Mean	Std Dev	Minimum	Maximum	Median	Lower quartile	Upper quartile	p-value
%Lymphocytes	PD	**CD25** ^+^ **FOXP3** ^+^	13	61.23	104.67	–64.15	335.29	35.92	4.11	85.29	**0.0425**
		**CD127** ^lo^ **CD25** ^+^	13	49.14	107.22	–39.91	393.33	23.71	10.94	46.04	**0.0215**
		**FOXP3** ^+^	13	58.29	101.01	–60.00	306.67	30.85	1.35	82.14	**0.0479**
		**FOXP3** ^+^ **CD25** ^hi^	13	76.23	116.15	–65.56	382.35	53.85	16.13	128.26	**0.0215**
		FOXP3^+^CD25^int^	13	56.24	109.46	–66.84	339.56	23.81	–8.78	66.67	0.1465
		FOXP3^+^CD25^–^	13	74.48	161.35	–52.22	525.00	15.38	–32.35	141.18	0.3396
	HD	CD25^+^FOXP3^+^	8	–2.59	30.86	–45.85	36.6	0.07	–28.34	22.54	0.8438
		CD127^lo^CD25^+^	8	3.79	33.83	–50.21	50.39	6.69	–19.98	28.34	0.7422
		FOXP3^+^	8	–2.63	26.98	–43.33	33.33	2.32	–24.02	16.18	0.9453
		FOXP3^+^CD25^hi^	8	12.94	53.05	–60	123.08	–1.39	–10.17	31.79	0.8125
		FOXP3^+^CD25^int^	8	–3.47	29.53	–51.27	37.98	3.7	–26.73	15.79	0.8438
		FOXP3^+^CD25^–^	8	–4.92	25.61	–47.22	22.86	1.1	–25	16.42	0.7422
